# The Clinical Spectrum of Mosaic Genetic Disease

**DOI:** 10.3390/genes15101240

**Published:** 2024-09-24

**Authors:** Hanabi Geiger, Yutaka Furuta, Suné van Wyk, John A. Phillips, Rory J. Tinker

**Affiliations:** Division of Medical Genetics and Genomic Medicine, Vanderbilt University Medical Center, Nashville, TN 37232, USA; hanabi.u.geiger@vanderbilt.edu (H.G.); yutaka.furuta@vumc.org (Y.F.); sune.van.wyk@vumc.org (S.v.W.); john.a.phillips@vumc.org (J.A.P.III)

**Keywords:** mosaicism, mosaic, heteroplasmy, X-linked lethal disease, chimera

## Abstract

Genetic mosaicism is defined as the presence of two or more cell lineages with different genotypes arising from a single zygote. Mosaicism has been implicated in hundreds of genetic diseases with diverse genetic etiologies affecting every organ system. Mosaic genetic disease (MDG) is a spectrum that, on the extreme ends, enables survival from genetic severe disorders that would be lethal in a non-mosaic form. On the milder end of the spectrum, mosaicism can result in little if any phenotypic effects but increases the risk of transmitting a pathogenic genotype. In the middle of the spectrum, mosaicism has been implicated in reducing the phenotypic severity of genetic disease. In this review will describe the spectrum of mosaic genetic disease whilst discussing the status of the detection and prevalence of mosaic genetic disease.

## 1. Introduction

**Figure 1:**
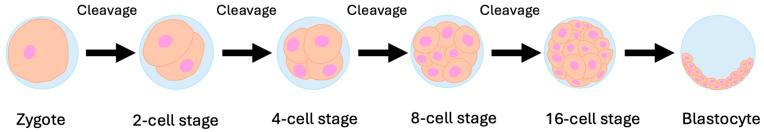
Stages of early embryogenesis: from fertilization to blastocyst formation. Early embryogenesis begins with fertilization, where a sperm and oocyte merge to form a zygote. The zygote undergoes rapid divisions (cleavage), forming smaller cells called blastomeres. As it reaches the 16–32 cell stage, the embryo becomes a compact morula. Around day 5, the morula develops into a blastocyst, consisting of an outer trophoblast (which will form the placenta) and an inner cell mass (which will become the embryo), along with a fluid-filled cavity.

**Figure 2:**
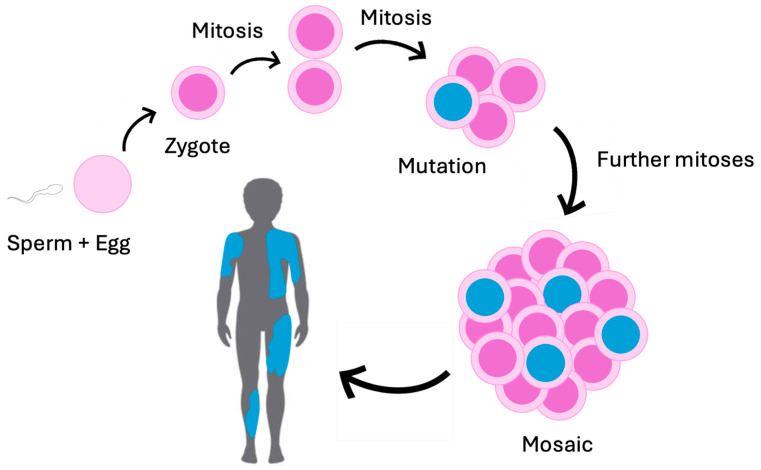
Schematic representation of mosaicism occurring when early zygotic mutations lead to distinct cell populations within the individual. Mosaicism arises when a mutation occurs during the mitotic division of a zygote, formed by the conjugation of sperm and egg. As the zygote undergoes further mitotic division, the mutated cells (in blue) continue to proliferate alongside normal cells (in purple), leading to the coexistence of genetically distinct cell populations within the individual.

**Figure 3:**
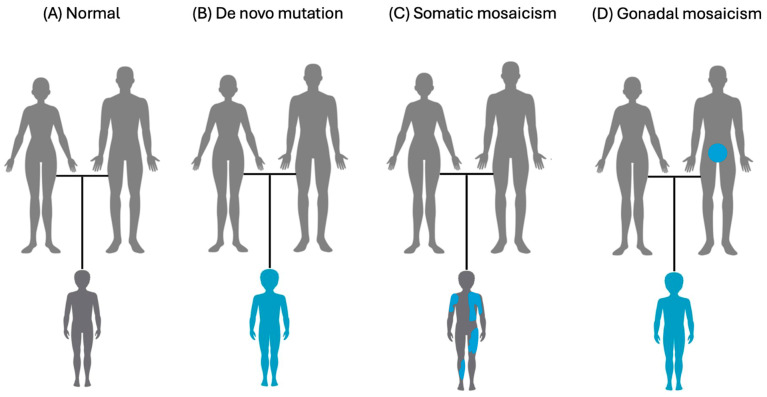
Types of genetic mutations and their roles in somatic and gonadal mosaicism. (**A**) Normal healthy individual without any genetics mutations or mosaicism. (**B**) De novo mutation: A genetic mutation arises in a single gonadal cell or in the zygote within the first few divisions in a germline mutation. The offspring may be heterozygous in every cell. (**C**) Somatic mosaicism: A mosaic mutation occurs in soma (non-gonadal) cells after fertilization. The offspring may have two genetically different cell lines, with some cells carrying the mutation and others not. (**D**) Gonadal mosaicism: A mosaic mutation arises exclusively in the gonadal cells (ess or sperm) of an unaffected parent. Offspring may be heterozygous in every cell.

**Figure 4:**
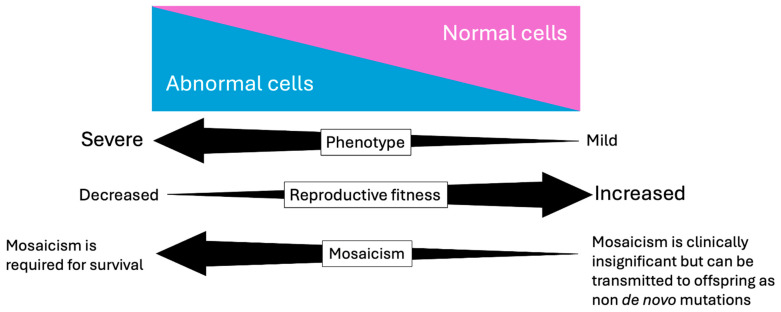
Spectrum of mosaic genetic diseases and their impact on phenotype and reproductive fitness. The spectrum of mosaic genetic diseases (MGD) ranges from severe conditions compatible with life due to mosaicism (**left**) to disorders with minimal phenotypic impact where mosaicism primarily affects reproductive cells (**right**). In severe cases, mosaicism is associated with high severity and low reproductive fitness. In intermediate cases, mosaicism reduces the severity of the phenotype. At the mild end, gonadal mosaicism has little effect on phenotype but may be transmitted to offspring as non de novo mutations in future generations, associated with high reproductive fitness.

Genetic mosaicism is the presence of two or more cell lines with different genotypes that arise from a single zygote (Figure 1). This definition helps to explain genetic mosaicism, where many cells with different genetic makeups exist together in one individual [1] (see Figure 2). Mutations accumulated throughout life make us all genetic mosaics to some extent [1]. Clinically, Mosaicism or Mosaic Genetic Disease (MGD) refers to a condition arising from mutations in early zygotic development [1]. This results in two or more genotypes in an individual due to separate cell lineages [1] (see Figure 2). Mosaicism is distinct from chimerism which is the uncommon state of having two or more populations of genetically distinct cells derived from two or more zygotes [2]. This is distinct from mosaicism whose different cell lines are derived from a single zygote [2]. Different cells can exhibit MGD due to changes ranging from single nucleotide variants (SNVs) to whole chromosome variants [1]. With such a broad range of variants and their effects, the spectrum of MGD needs classification [1]. Broadly, MGD can result from mosaicism in somatic (non-gonadal), gonadal, or both cell types (see Figure 3). This is an important distinction because pathogenic Parental Gonadal Mosaicism (PGM) can lead to abnormal non-mosaic genotypes in offspring, which may be misattributed as the result of a de novo mutation [3]. Segmental and nonsegmental somatic mosaicism can occur in MGD. For example, Blaschko lines showing different skin pigmentation patterns can result from mosaicism and can be useful in identifying mosaicism [4]. We can consider MGD to be a continuum from lethal disorders to those that are compatible with life due to mosaicism on the extreme ends of the spectrum. These disorders can be associated with highly severe phenotypes and low reproductive fitness. In the middle of the spectrum is mosaicism, which can reduce the severity of the phenotype (Figure 4). At the mildest end of the spectrum are gondola mosaic disorders, where mosaicism has little if any impact on phenotype but is a source of pathogenic variants in the gonads that can be transmitted to produce heterozygous affected offspring. This end of the spectrum is associated with high reproductive fitness. The mechanisms that give rise to mosaicism are important to understanding the impact of MGD on affected individuals and the differences seen in their phenotypes. In this article, we will explore the spectrum of MGD. Additionally, we will discuss relevant technologies to detect MGD, including Next Generation Sequencing (NGS). We will highlight potential implications of MGD to genetic research, diagnosis, and therapy. While mosaicism often causes cancer and clonal hematopoiesis, these will not be discussed in this review, which will focus on clinical mosaicism in Mendelian genetic disease [5,6].

## 2. Biological Origins of Genetic Mosaicism

Mosaicism results from a change in a cell’s DNA sequence, copy number, or methylation status during development [7]. These changes can arise from a variety of mechanisms including inheriting mutations from parents with gonadal mosaicism, as well as reversion, or nondisjunction [7]. Preimplantation studies have shown that anaphase lag during cell division is the main cause of mosaic aneuploid in embryogenesis [8]. Mosaicism is often found in genes that have an increased risk of mutations [9]. Factors that contribute to this increased risk include large genes that have many exons and introns that provide more targets for mutations [9]. Such changes can perturb splice site mutations, repeated DNA segments that can misalign during replication, and methylated regions, which can predispose to C > T and G > A transitions in methylated CG dinucleotides in some but not all of an individual’s cells during early mitosis [8,9].

## 3. The Most Severe End-of-Spectrum Diseases Where Mosaicism Is Required for Survival

Some genetic diseases exist only in mosaic form because the non-mosaic genotype is lethal [9]. The presence of two distinct genotypes in different cells of MGD individuals enables some cells to have a normal phenotype while others express a pathogenic trait [9]. With a smaller percentage of cells being pathogenic, the disease is typically milder than in non-mosaic individuals, in whom all the relevant cells are pathogenic [9]. McCune Albright syndrome, for example, is characterized by a *GNAS* mutation that is lethal, and is only seen in mosaic individuals who have a survivable phenotype [4]. The presumed mechanism is an early somatic gain of function mutation that arises in a cell whose daughter cells do not perturb essential functions [4].

For individuals with karyotypic abnormalities, mosaicism can significantly influence the phenotypic effects [10]. Chromosomal Trisomies arise from nondisjunction during cell divisions [10]. Only three trisomies (13, 18, 21) are compatible with life in non-mosaic forms [11]. Outside of these three small autosomes (non-sex chromosomes), mosaicism is essential for the survival of trisomies of all the remaining autosomes. For example, Trisomy 8 is typically a lethal condition; however, in the case of Mosaic Trisomy 8, those with a large percentage of non-trisomic cells can be mildly affected or even asymptomatic [10,12]. Thus the severity of the problems can vary depending on the ratio of normal to trisomy cells [10] (see Figure 4). Likewise, Trisomy 9 can only exist as a mosaic form, since having only trisomy cells results in miscarriage [13]. However, even infants with mosaic Trisomy 9 typically die by 9 months [13]. The alternative result of chromosomal nondisjunction is monosomy which is lethal because only a single copy of an autosome is retained and monosomy cells are incapable of producing adequate number of proteins and ribosomes for survival [13,14]. For this reason, the complete monosomy of autosomes is not compatible with life, but in rare cases, mosaic monosomies have allowed for the survival of individuals. Case studies have found mosaicism to be a mechanism of survival in monosomy of chromosomes 2, 4, 5 (partial), 6, 7, 8 (partial), 9 (partial), 10 (partial), 11 (partial), 12, 13, 14, 18, 20, 21, and 22 [14,15,16,17,18,19,20,21,22,23,24,25,26,27].

Another mechanism by which mosaicism can reduce the pathogenic effects of lethal genetic disorders is seen in X-linked (XL) lethal conditions [9]. Many XL disorders are typically lethal in males because they lack a functional copy of the XL gene to produce a functional protein [7]. However, in males with MGD, sufficient normal cells can prevent disease despite the presence of abnormal cells [7]. Incongentia Pigmenti (IP) is an XL disorder characterized by skin lesions and neurological problems serve as an example of this [7]. A mechanism for the survival of males with IP is somatic mosaicism, which results in only some of their X chromosomes having a mutant *IKBKG* gene [7]. Similarly, Rett syndrome, another XL dominant disorder, is usually lethal in males without MGD [28]. Rett syndrome is a neurological disorder that arises due to mutations in the XL *MECP2* gene [28]. Females, including those with Rett syndrome, have two X chromosomes [28]. All female cells have inactivation of one of their X chromosomes called X-inactivation [28]. If females heterozygous for an abnormal *MECP2* gene have skewed X inactivation so that few of the cells express the abnormal gene, they can be less affected or penetrant for RETT syndrome [28]. However, since males have only one X chromosome, mosaicism rather than skewed X inactivation is essential for them to express functional *MECP2* genes and survive with a *MECP2* variant [28].

A list of commonly known disorders where mosaicism is essential for survival can be found in Table 1.

## 4. Mosaicism Resulting in a Potentially Milder Phenotype

Mosaicism can cause a milder phenotype of genetic disease resulting from a pathogenic mutation [9] (see Figure 2 and Figure 4). If a severe mutation occurs in the zygote, a low level of mosaicism for the mutation or its presence in only non-susceptible organs may produce a milder phenotype [9]. In some cases, the mutation may revert to wild-type in some cells but not all and cause mosaicism [9]. For example, spontaneous genetic reversion can correct certain Fanconi anemia (FA) mutations. This leads to mosaic Fanconi anemia which exhibits a milder phenotype because early developmental repair reducethe proportion of cells affected by the mutation [9]. Other disorders such as Wiskott–Aldrich syndrome and adenosine deaminase deficiency have also been associated with mosaic repair leading to milder phenotype [9]. Altogether, mosaic repair has been implicated in at least 12 distinct pathologies [9]. Turner Syndrome (TS) (45,X/46,XX mosaicism) is caused by a partial or complete deletion of one of the two X chromosomes that are normally present in females [29]. Of adults with Turner syndrome, 50% have been implicated in having a mosaic phenotype, resulting in a milder phenotype. However, it has been postulated that all surviving individuals with Turner syndrome were or still are mosaic to some extent [29].

Mosaicism frequently manifests in the brain due to the complexity of cell lineages involved in neural development. Here, MGD frequently results in milder phenotypes than non-mosaic forms of neurogenic disease. The accumulation of mutations during this process can result in focal MGD phenotypes in the brain. For example, the mTOR pathway is critical in neural differentiation and metabolism [45]. Focal Cortical Dysplasia (FCD) is a spectrum of diseases that can be linked to MGD of this pathway [43]. Specifically, mutations in this pathway are responsible for between 50–60% of all hemimegaloencephaly (HME) and FCD2 [43]. Gain-of-function mutations in mTOR activators can result in organizational errors as well as abnormal cells [46,47]. Two-hit (one germline and one somatic) loss of function mutations in repressors of the mTOR pathway have similar outcomes and can result in tuberous sclerosis complex [43]. It is thought that these mutations that produce mosaicism occur after the left/right axis in cortical progenitors has occurred [43]. The greater the proportion of cells that carry a pathogenic mutation increases the probability that the disease will be more severe [43]. Genome Sequencing (GS) has shown a connection between autism spectrum disorder (ASD) and cerebral MGD [48]. Mosaic missense mutations in genes associated with ASD risk have been detected in 0.8–1.3% of individuals with ASD [48]. This is a higher incidence than would be expected and it is postulated that MGD may cause a 3.4% increased risk of development of ASD [48]. The cooccurrence of ASD and mosaicism was found to be around 22% [49]. Mosaicism is likely to be common in the brain as it has been shown that at birth there are already 500–1000 SNVs per neuron [45]. In early human embryos, the rate of accumulation is approximately 1.3 SNVs per cell division, and this increases during neurogenesis and then drops off postnatally [45]. These high rates of SNV accumulation suggest that brain mosaicism is likely common and identifying its connections to pathogenic phenotypes may be fruitful. The investigation of the repair mechanisms of these SNVs suggests that deficiencies in single and double-strand break (SSB, DSB) repair and nuclear excision resultingly leads to mosaic immunodeficiency and neurological disorders [45]. Specifically, NER has been found in Cockayne syndrome, Xeroderma Pigmentosum, neurodegeneration, and microcephaly, with cortical neurons especially affected [45].

**Figure 5:**
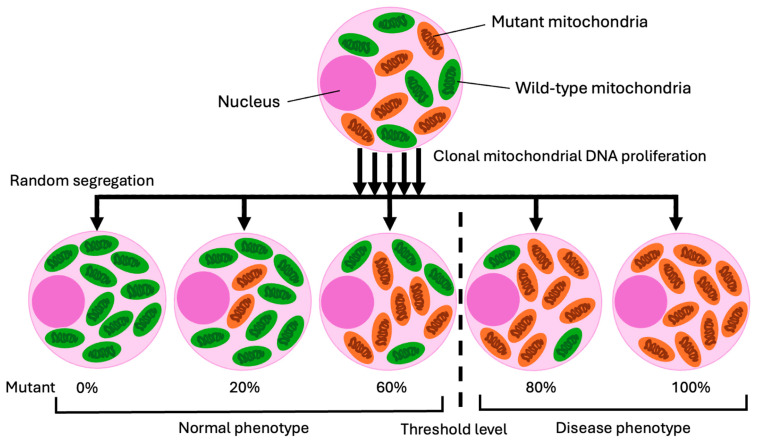
Replicative segregation of heteroplasmic mitochondrial DNA mutations. As mitochondrial DNA replicates, the mitochondria undergo fission and fusion, leading to the random distribution of mutant and wild-type DNA into daughter cells, which may result in varying proportions of each. A parent cell with low-level mutant mitochondrial DNA heteroplasmy can produce progeny with varying heteroplasmy levels, potentially exceeding the threshold for expressing a mutant phenotype after replication. Mitochondrial DNA mutations, often heteroplasmic, can coexist with wild-type mitochondrial DNA in the same cell. A pathogenic mutation generally requires a heteroplasmy level above 80% to surpass the biochemical threshold.

Heteroplasmy is a specific form of mosaicism where there is the presence of more than one type of mitochondrial DNA (mtDNA) in the mitochondria of a single individual [50]. This can occur because there are many mitochondrial strands in the cytoplasm, unlike the diploid chromosomes in the nucleus of cells (see Figure 5) [37]. The first cause of heteroplasmy is thought to be mtDNA mutations that occur over time [37]. The second is the inheritance of heteroplasmy from the mother, wherein an already heteroplasmic oocyte gives rise to a zygote with multiple mtDNA variants (see Figure 5) [50]. Heteroplasmy has been implicated in the development and expression of various mitochondrial disorders [50]. Mitochondrial disorders stem from the mitochondria’s central role in metabolism and dysfunction, which results in defective energy production, poor growth, and developmental delays [51,52]. Importantly, the proportion of mutant to normal mtDNA alleles within cells correlates with the severity and onset of these disorders, with higher proportions of mutant mtDNA resulting in more severe phenotypic manifestations [51]. Over 500 mtDNA mutations have been implicated in dozens of mitochondrial conditions [38].

Epigenetics refers to changes in gene expression and regulation that do not involve alterations to the underlying genetic code [53]. Epigenetic mosaicism occurs when pathogenic modifications affect gene expression in only a proportion of an individual’s cells [53]. This phenomenon is particularly challenging to detect due to the natural presence of diverse epigenotypes across different cells in the body, making us all epigenetically mosaic to some extent [53]. The clinical significance of epigenetic mosaicism lies in the pathogenic outcomes that arise when epigenetic regulation deviates from the norm. Epigenetic mosaicism was described in several genomic imprinting disorders, including hydatidiform mole, Angelman syndrome (AS), Prader–Willi syndrome (PWS), Silver–Russell syndrome (SRS), Beckwith–Wiedemann syndrome (BWS), Temple syndrome, pseudohypoparathyroidism 1B (PHP1B), and transient neonatal diabetes mellitus (TNDM) [53]. Unlike a non-mosaic situation, not all the corresponding genes are epigenetically silenced, resulting in a milder phenotype [53]. Further work is needed to describe the phenotypic impact of mosaic epigenetic disease.

## 5. Gonadal Mosaicism

At the far end of the spectrum is gonadal mosaicism, which typically leaves individuals unaffected but creates a risk of transmitting pathogenic variants to their offspring [3]. Gonadal mosaicism arises from meiotic errors in gametogenesis, especially in males as they age [54]. These errors produce a population of mosaic germ cells which differ from the somatic cells [55]. Recent research on non-mosaic patients presenting with seemingly *de novo* mutations demonstrate that some of these presumed *de novo* mutations were in fact transmitted from a presumed asymptomatic parent who had undetected gonadal mosaicism [3]. The prevalence of this parental gonadal mosaicism (PGM) as the actual cause of “*de novo*” mutations in an affected child is reported to range from 2.17 to 20% [3,56]. Focusing on neurodevelopmental disorders, 3.7% of “*de novo*” offspring were found to have mosaic parents [57]. These values are significant, as studies have found *de novo* mutations to be responsible for 80% of pathogenic variants implicated in developmental disability, and, more broadly, they are believed to be responsible for 65% of all pathogenic variants [58,59]. With such a high prevalence, even a 2% misattribution becomes relevant for genetic counseling. This provides insights into how PGM can explain why presumed unaffected parents can have more than one affected child [55]. In PGM, the risk of recurrence in offspring is presumed to be low but theoretically could be as high as 50% [60]. While PGM contributes to at least 24 disorders theoretically, any genetic disorder could arise from this type of transmission [9,55]. Please see Table 1 for a list of disorders that have been implicated in gonadal mosaicism.

## 6. Prevalence of MGD

The prevalence of MGD is poorly understood due to the difficulty of detection of MGDs [30]. Most research on the prevalence of MGD is from embryo data that lacks validity when studying human genetic disease. Studies of in vitro fertilization (IVF) have shown that 13.9% of preimplantation embryos have mosaic aneuploidies [61]. Interestingly, 5.4% of human blastocysts were found to be mosaic, but these numbers are variable through development, ranging from 15% to 90% in studies of cleavage-stage human embryos [62]. These values are not representative of that seen in the adult population because mosaicism in embryos often results in miscarriage [62]. An understanding of the prevalence of mosaicism beyond gestation, particularly in the context of MGD, has been gained through the analysis of patients in the Undiagnosed Disease Network using Next-Generation Sequencing (NGS) [3]. This analysis revealed that 4.5% of subjects were mosaic, with three-quarters having mosaicism of nuclear genes and the others were divided between chromosomal and mitochondrial mosaicism [3]. A broader review, which analyzed Vanderbilt’s electronic health records for genetic diagnoses, presented a slightly different ratio, indicating a higher prevalence of mitochondrial mosaicism compared to nuclear mosaicism [3]. Population-wide studies investigating the rate of mosaicism in the general population showed prevalence rates of nuclear mosaicism at 0.74% [63]. This suggests that mosaic genes could potentially be enriched in those with genetic disease. Additionally, age was positively correlated with the prevalence of mosaic chromosome abnormalities [63]. When exome sequencing (ES) was used to analyze a cohort of children with severe developmental disorders, a ~3% rate of post-zygotic mosaicism was seen [64]. A similar cohort found 11 structural mosaic events (in 9 patients) out of a sample size of 4911 [65]. The evolving technology to detect mosaicism is partly responsible for the inconsistency in these values. The continued improvement of technology and understanding of mosaicism as a whole is needed to better understand mosaic prevalence.

## 7. Technology

The increase in mosaicism research and understanding has largely been driven by technological advancements in detecting and analyzing mosaicism. Next-generation sequencing (NGS), which includes ES and GS, has significantly improved the detection of MGD caused by SNVs by enabling the efficient testing of many gene copies through increased read depth which is crucial in detecting MGD [66]. Previously, detecting mosaicism at levels < 30% was challenging, but with the advent of NGS technology, mosaicism can now be reliably identified at progressively lower levels as the read depth increases [66]. Additionally, the digital outputs from NGS provide better sensitivity in detecting variants and MGD than Sanger sequencing [66]. However, the impact that long-read sequencing will have in detecting mosaicism remains unclear at present. With the growth in the detection of MGD, further work is needed to catalog these changes systematically. MosaicBase.com is a knowledge base that catalogs mosaic mutations associated with Mendelian diseases [67]. This rapidly growing database has already amassed 34,689 validated mosaic variants, manually compiled from 383 publications, and continues to expand [67]. Further efforts are required to structure information on mosaic variants and their impact on phenotype, survival, and transmission risk.

## 8. Conclusions

Research on mosaicism has provided insights into the origins, effects, and contributions of mosaicism to the spectrum of MGD. MGD is not a binary phenomenon but a spectrum that modulates the risk of transmission as well as the severity of genetic disease with diverse genetic etiologies. The improved ability to detect MGD has increased the understanding of its causes and has broader implications for the scientific community and medicine as a collective. However, while substantial gaps in our understanding of the full scope and severity of mosaicism’s effects remain, it is almost certain that our knowledge, insights, understanding, and research and clinical applications will expand considerably.

## Figures and Tables

**Table 1:** A table demonstrating the different types of mosaicism with example diseases.

Type of Mosaicism	Description	Example Disease	Reference
Mosaicism required for the survival of aneuploidies	Only three non-mosaic trisomies—13, 18, and 21—are compatible with life; other trisomies are non-viable unless present in a mosaic form.	Trisomy 8, 9, 13, 18, and 21 Tetrasomy 12p (Pallister-Killian syndrome) Monosomy: X, 2, 4, 5 (partial), 6, 7, 8 (partial), 9 (partial), 10 (partial), 11 (partial) [20], 12, 13, 14, 18, 20, 21, and 22	[14,15,16,17,18,19,20,21,22,23,24,25,26,27,29,30,31,32]
Mosaicism modulating an X-linked lethal phenotype	Mosaicism affecting the sex chromosomes with varying effects depending on the sex of the individual.	Chondrodysplasia punctata 2 Dent disease Fabry disease Incontinentia pigmenti Oral-facial-digital syndrome type I Rett syndrome	[33,34]
Mosaicism facilitating survival of a lethal non-mosaic genotype	Mosaicism which can cause a milder phenotype due to certain repair mechanisms to improve survivability despite mutations.	Adenosine deaminase deficiency Bloom syndrome Dyskeratosis congenita Diamond–Blackfan anemia Epidermolysis bullosa Fanconi anemia Turner syndrome Severe combined immunodeficiency Tyrosinemia Wiskott–Aldrich syndrome	[35,36]
Mitochondrial heteroplasmy	When an individual has a fraction of their cells with multiple distinct sets of mitochondrial DNA.	Mitochondrial diseases	[37]
Epigenetic mosaicism	Typically related to postzygotic methylation abnormalities, epigenetic mosaicism relates to changes in the way genes are expressed rather than the DNA itself.	Angelman syndrome Beckwith–Wiedemann syndrome Kagami–Ogata syndrome Prader–Willi syndrome Pseudohypoparathyroidism 1B Silver–Russell syndrome Temple syndrome Transient neonatal diabetes mellitus	[38,39,40]
Somatic (non-gonadal) mosaicism	Mosaicism affects the somatic cells of the body (not the germ cells).	Overgrowth *PIK3CA*-related overgrowth spectrum PTEN hamartoma tumor syndrome RASopathies Neurodegenerative Alzheimer’s disease Ataxia-telangiectasia Monogenic McCune–Albleft syndrome Sturge–Weber syndrome Cancer predisposition syndromes von Hippel-Lindau disease	[39,41]
Gonadal mosaicism	Mosaicism affecting the germline cells. Gonadal mosaicism can pass down mutations from a mosaic parent to a non-mosaic offspring and be misinterpreted as de novo mutations.	Autosomal dominant Achondroplasia Neurofibromatosis type 1 Osteogenesis imperfecta Tuberous sclerosis X-linked Duchenne muscular dystrophy Hemophilia A and B	[1,41,42]
Brain	Mosaicism focally affecting the brain and originates during brain development.	Autism spectrum disorder Focal Cortical Dysplasia Hemimegaloencephaly Tuberous sclerosis Primary aldosteronism, seizures, and neurologic abnormalities	[43,44]
